# Comparison of different virtual chromoendoscopy classification systems for the characterization of colorectal lesions

**DOI:** 10.1002/jgh3.12382

**Published:** 2020-07-07

**Authors:** Leonardo Zorron Cheng Tao Pu, Takeshi Yamamura, Masanao Nakamura, Doreen S C Koay, Amanda Ovenden, Suzanne Edwards, Alastair D Burt, Yoshiki Hirooka, Mitsuhiro Fujishiro, Rajvinder Singh

**Affiliations:** ^1^ Faculty of Health and Medical Sciences The University of Adelaide Adelaide South Australia Australia; ^2^ Department of Gastroenterology and Hepatology Nagoya University Nagoya Japan; ^3^ Department of Endoscopy Nagoya University Hospital Nagoya Japan; ^4^ Department of Gastroenterology Lyell McEwin Hospital Adelaide South Australia Australia; ^5^ Department of Gastroenterology and Gastroenterological Oncology Fujita Health University Toyoake Japan

**Keywords:** adenoma, colonoscopy, colorectal neoplasms, serrated polyp

## Abstract

**Background and Aim:**

Commonly used classifications for colorectal lesions (CLs) include the Narrow Band Imaging (NBI) International Colorectal Endoscopic (NICE) and Japan NBI Expert Team (JNET) classifications. However, both lack a sessile serrated adenoma/polyp (SSA/P) category. This has been addressed by the modified Sano's (MS) and Workgroup serrAted polypS and Polyposis (WASP) classifications. This study aims to compare the accuracy of wNICE and wJNET (WASP added to both) with the stand‐alone MS classification.

**Methods:**

Patients undergoing colonoscopy at an Australian tertiary hospital who had at least one CL detected were prospectively enrolled. In the exploratory phase, CLs were characterized in real time with NBI and magnification using all classifications. In the validation phase, CLs were assessed with both NBI and Blue Laser Imaging (BLI) by four external endoscopists in Japan. The primary outcome was the comparison of wJNET and MS. Secondary outcomes included comparisons among all classifications and the calculation of interrater reliability.

**Results:**

A total of 483 CLs were evaluated in real time in the exploratory phase, and four sets of 30 CL images (80 on NBI and 40 on BLI) were scored in the validation phase. For high‐confidence diagnoses, MS accuracy was superior to wJNET in both the exploratory (86% *vs* 79%, *P* < 0.05) and validation (85% *vs* 69%, *P* < 0.05) phases. The interrater reliability was substantial for all classifications (*κ* = 0.74, 0.69, and 0.63 for wNICE, wJNET, and MS, respectively).

**Conclusions:**

MS classification achieved the highest accuracy in both the exploratory and validation phases. MS can differentiate serrated and adenomatous polyps as a stand‐alone classification.

## Graphical abstract

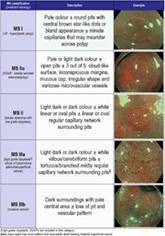

Many classifications have been propose to characterize colorectal lesions but few include SSA/Ps.Comparison between classifications that have this potential lack in the literature.This study compares the WASP+NICE, WASP+JNET and MS classifications with NBI and BLI imaging.

## Introduction

Screening programs based on fecal tests and colonoscopy have been implemented to tackle the scourge of colorectal cancer (CRC). The efficacy of such programs relies on the detection of CRC precursors. Initially, screening programs were specifically designed to detect and remove adenomatous polyps, which have been thought for decades to be the sole precursors leading to CRC. However, the appearance of “missed” CRCs promoted a search for other explanations, and the role of serrated polyps in CRC carcinogenesis emerged.

Sessile serrated adenomas/polyps (SSA/Ps) have been shown to contribute to up to a third of CRCs, contrasting with the fact that they have a low reported prevalence in both the East and West.^1^, ^2^ The discrepancy between the prevalence of SSA/Ps and their share of responsibility for CRC may be explained by the fact that the serrated pathway has a higher risk of developing CRC than the traditional adenoma–carcinoma pathway.^3^ This may be due to a more “aggressive” pathophysiology.^4^ However, it is also possible that the difficulty in detecting and characterizing SSA/Ps (misdiagnosing it to be nonneoplastic) could be one of the reasons for its low prevalence.

Although image‐enhancing endoscopy technologies have proven to be effective in identifying and discriminating adenomatous polyps from other colorectal lesions (CLs), the differentiation of serrated lesions is more challenging. Hyperplastic polyps (HPs) are usually considered to be benign and could potentially be left *in situ* when they are smaller than 5 mm and are restricted to the rectosigmoid region.^5^, ^6^ A meta‐analysis showed that, despite promising results with narrow‐band imaging (NBI), more data are needed to confirm the use of image‐enhancing endoscopy as a useful tool for SSA/Ps.^7^ Nevertheless, NBI appears to be the most promising technology for this and has met the thresholds of the American Society for Gastrointestinal Endoscopy (ASGE) Preservation and Incorporation of Valuable endoscopic Innovations (PIVI) program.^5^

The use of NBI has been studied by several experts with a variety of classifications, including the Sano, Modified Sano's (MS), Hiroshima, Japan NBI Expert Team (JNET), Showa, Jikei, NBI International Colorectal Endoscopic (NICE), and Workgroup serrAted polypS and Polyposis (WASP) classifications. JNET (Figure S1, Supporting information) has been recently proposed in Japan as an amalgamation of all Japanese classifications.^8^ NICE (Figure S2) is still one of the most widely used classifications (especially in the West), probably due to its simplicity and practicality. However, recently, MS was found to outperform the NICE classification.^9^ The MS classification was conceived in 2013 and consists of five categories (I, IIo, II, IIIa, and IIIb), while JNET has four (1, 2A, 2B, and 3), and NICE has three.^1^, ^2^, ^3^ Of all these classifications, only WASP and MS are able to classify SSA/Ps into a separate category.^8^, ^9^, ^10^, ^11^, ^12^, ^13^, ^14^ Other classifications assign SSA/Ps alongside HPs, hence mixing neoplastic with nonneoplastic polyps. As the role of the serrated pathway becomes clearer, the use of an endoscopic classification that could characterize SSA/Ps is important. The use of endoscopic classifications that cannot differentiate HPs from SSA/Ps might lead to the decision of leaving a neoplastic polyp that can contribute to interval CRC.

The aim of this study was to compare the accuracy of the three endoscopic classifications with the ability to differentiate serrated polyps: NICE and JNET combined with WASP (wNICE and wJNET, respectively) and the MS classification.

## Methods

Patients undergoing an elective colonoscopy at the Lyell McEwin Hospital, South Australia (August 2016 to January 2018), were prospectively enrolled in the ‘exploratory phase’. All procedures were performed by an expert in image‐enhancing endoscopy (RS), with over 10 years of experience in advanced imaging, using the Olympus® 190 series (Exera III) colonoscopes. Patients under 18 years of age; those undergoing emergency colonoscopy; pregnant women; and those with total colectomy, a previous or new diagnosis of inflammatory bowel disease (IBD), with no CLs identified, with familial adenomatous polyposis syndrome or Peutz‐Jeghers syndrome were excluded, as were those unwilling to participate. In addition, polyps that were detected and resected but not confirmed by histology (i.e. normal mucosa, melanosis coli or not retrieved) and patients who could not have a colonoscopy completed (e.g. poor bowel preparation) were excluded.

After a CL was detected with white light, NBI with magnification was used to characterize the lesion. This was performed with the aid of a transparent soft distal attachment cap (©Olympus D201). Two endoscopists (the endoscopist who was performing the procedure and a colleague) evaluated the characteristics of all CLs in real time during the procedure. These included size; Paris classification; serrated features; and JNET, MS, and NICE classifications. Serrated features included the four characteristics described by the WASP classification (Figure S3) in addition to two other features—varicose microvascular vessels (VMV) and presence of a mucous cap as per the MS classification. The utilization of WASP as a workup to characterize SSA/Ps for both JNET (from types 1 and 2A) and NICE (from types 1 and 2) was based on the original WASP publication.^13^ All polyps found during the study were removed for histopathological analysis.

**Figure 1:**
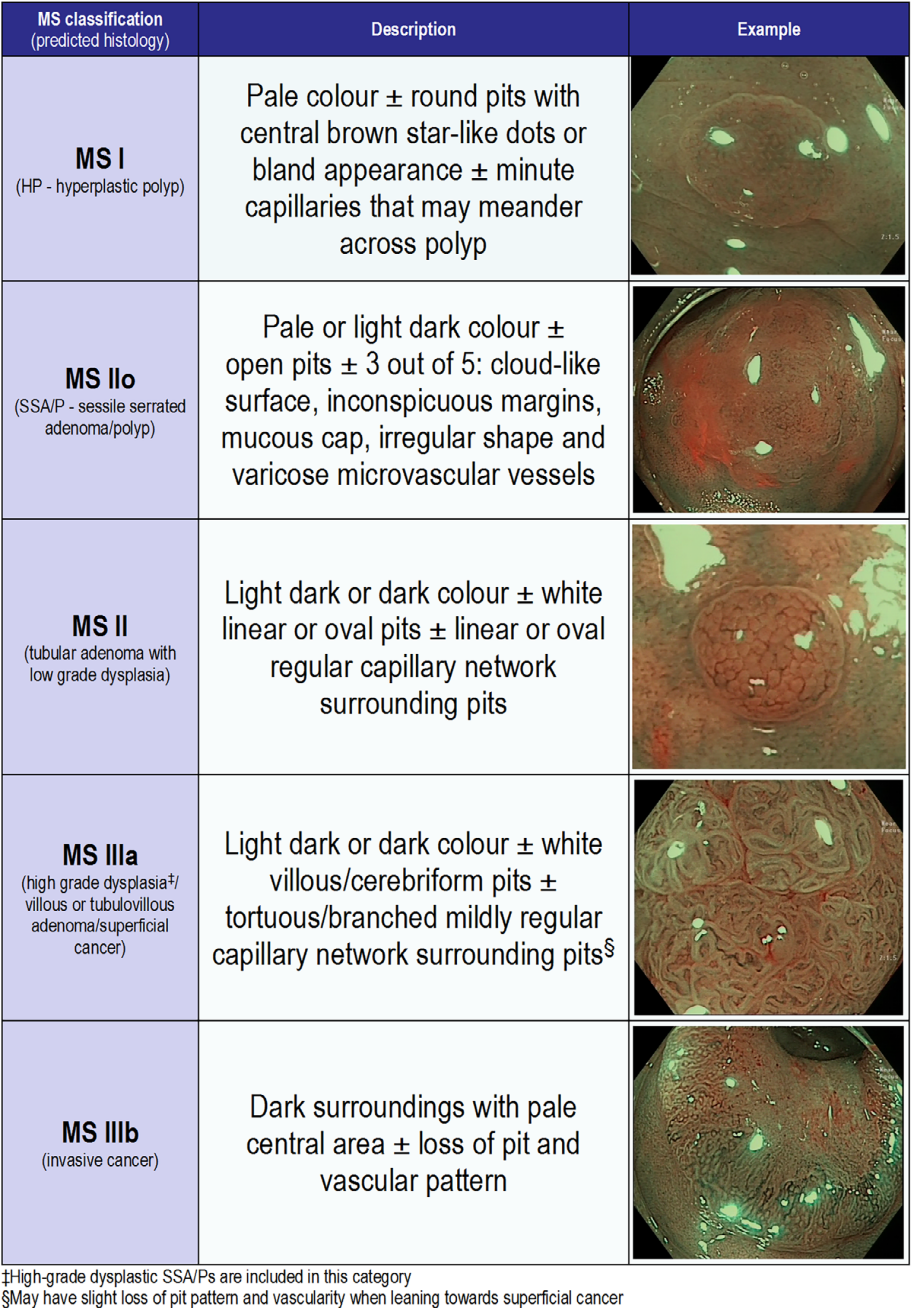
Modified Sano classification (adapted from Pu *et al*.^9^).

**Figure 2:**
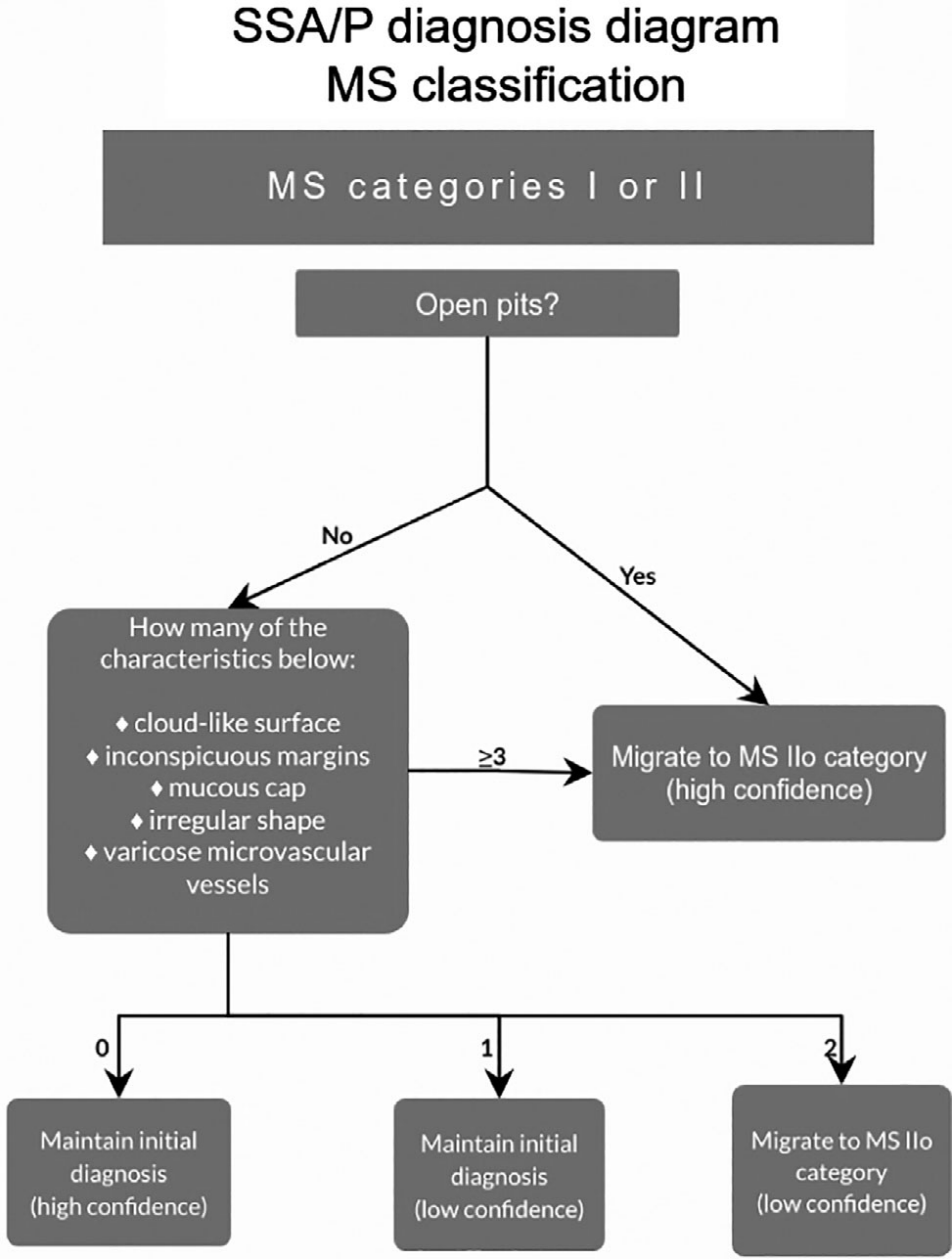
Sessile serrated adenoma/polyp diagnosis diagram for the modified Sano classification.

For diagnoses made with high confidence, there had to be agreement between both the endoscopists (RS and LZCTP). Although no specific training was given for this study, both were familiar with all classifications prior to the study. After both endoscopists were content that enough visualization with magnified NBI has been performed on the lesion, they would call the predicted types for each classification. If the predicted types matched, it would be termed a high‐confidence diagnosis. If not, the prediction of the senior endoscopist (RS) prevailed as low confidence. For wNICE types 1 and 2 and wJNET types 1 and 2A, the initial classification diagnosis was converted into SSA/P if two or more of the WASP features were found. For the MS classification categories I and II, the confidence level was also based on the serrated features, which were detected (Fig. 1). ‘Open pits’ feature was considered to be a high‐confidence feature by itself (i.e. independent SSA/P feature). The remaining five serrated features were considered interdependent SSA/P features, and their definition were as follow: MS I with high confidence for HP if “NO” for any serrated features; MS I with low confidence for HP if up to one “YES” for interdependent serrated features; MS IIo with low confidence for SSA/P if “YES” for two interdependent serrated features; and MS IIo with high confidence for SSA/P if “YES” for open pits or at least three “YES” for interdependent serrated features. This decision tree has been illustrated in a diagram for easier understanding (Fig. 2). All diagnoses were compared with the final histopathological report. In our institution, all polyps are evaluated by a general pathologist who seeks the input of a specialist gastrointestinal pathologist only if uncertain of the diagnosis. The criteria for diagnosis of SSA/Ps was based on the World Health Organization recommendations and consisted of at least two of the following criteria: (i) crypt dilation, (ii) irregularly branching crypts, and (iii) horizontally arranged basal area crypts at the basal (inverted T and/or L‐shaped crypts).^15^ Neoplastic lesions were considered to be any CL that had the potential or had already evolved into a CRC (i.e. tubular adenoma, tubulovillous adenoma, villous adenoma, traditional serrated adenoma, SSA/P, superficial adenocarcinoma, or invasive adenocarcinoma). The differentiation of high‐grade dysplasia (HGD), superficial cancer, and invasive cancer was adopted as limits for the severely dysplastic cells at the muscularis mucosae, 1000 μm into the submucosa, and the muscularis propria, respectively.^16^, ^17^ CLs of 5 mm or less were considered diminutive in size.

**Figure 3:**
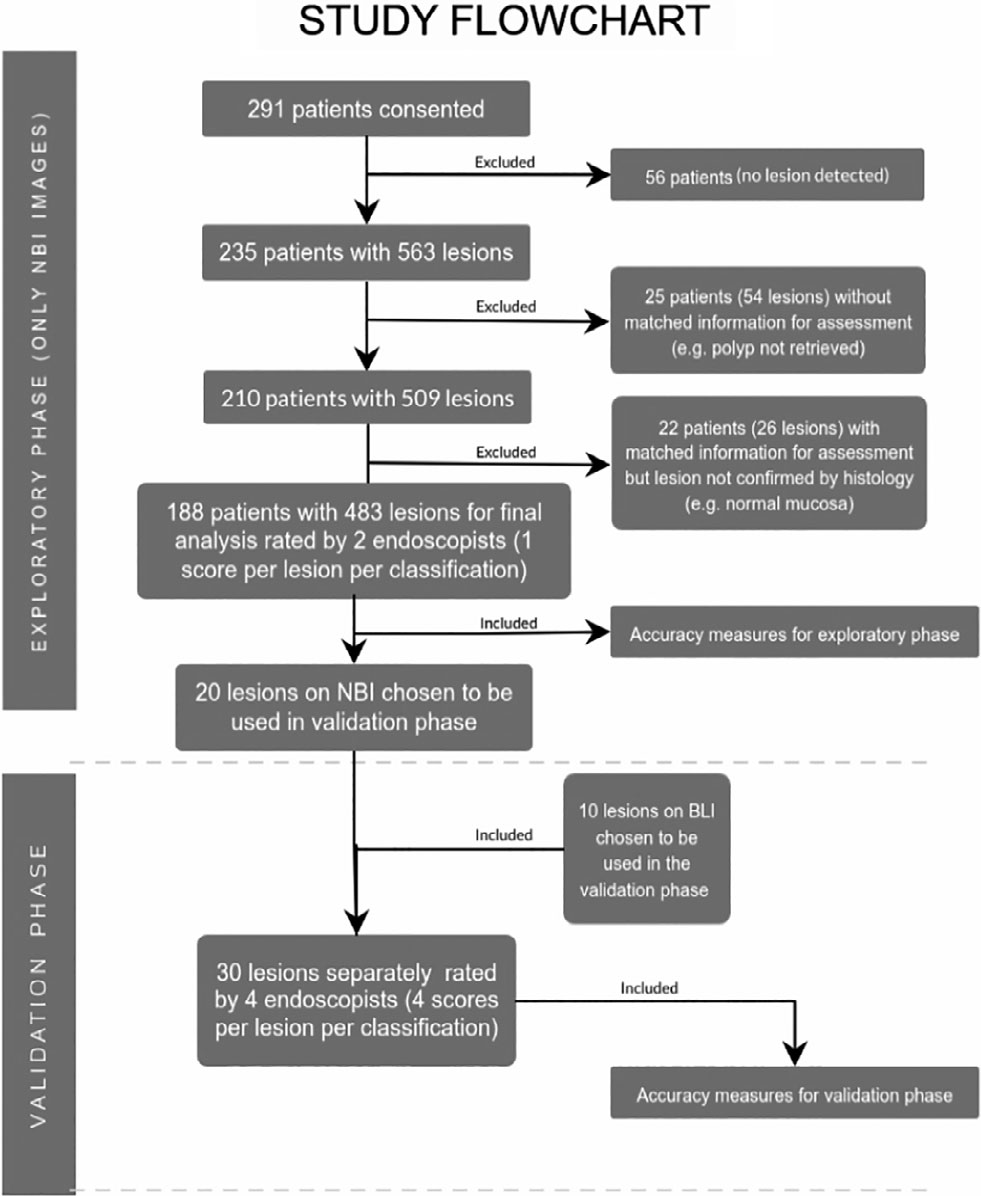
Study flowchart.

After all data from the exploratory phase of the study had been collected, 20 CLs' NBI‐magnified images were chosen from the Australian database. These were selected in order to be representative of all histological classes and varied in size (half ≤5 mm and half ≥10 mm). Ten additional images were collected from the Nagoya University Hospital electronic database and correlated with histology. These 10 images were captured by a Fujifilm 600 series colonoscope (©Fujifilm Corporation Japan), with BLI and magnification. In the validation phase, four experienced endoscopists (more than 5 years of experience with advanced imaging and magnification and part of the lower gastrointestinal endoscopy unit) were invited to participate in a 60‐min session. The study was explained, with emphasis on how to use all classifications. The four endoscopists selected for the validation phase had no clinical experience and little knowledge of the MS classification prior to the study but were familiar with the NICE, WASP, and JNET classifications. The design of the study has been summarized in a flowchart for better understanding (Fig. 3).

The primary outcome was the comparison of high‐confidence accuracy for wJNET and MS (five‐type classifications). Secondary outcomes included comparison of two‐type classifications (i.e. dichotomy of neoplastic *vs* nonneoplastic with wNICE, wJNET, and MS), four‐type classifications (i.e. wNICE, merged wJNET, and merged MS), an external validation of the classifications with NBI and BLI images, and subanalysis of specific datasets (i.e. high‐confidence accuracy, lesions ≤5 mm, lesions on NBI, and lesions on BLI). As wNICE is a four‐type classification and wJNET and MS are five‐type classifications, the two adenoma categories in wJNET and MS were merged into a single category (2A + 2B and II + IIIa, respectively) when compared to wNICE.

The sample size was calculated based on number of CLs against the primary outcome for the exploratory phase. An estimated sample size of 423 CLs would be required to have an 80% power with an alpha error of 0.05 to appreciate an increment of 6% in the prediction of histology with the MS classification (from 86% to 92%). This increment was inferred to be slightly lower than what was found in our previous study for the comparison of MS *versus* NICE.^9^ A McNemar test was used for comparison of accuracies for dichotomic classifications in the exploratory phase, two by two. Comparison of proportions was carried out with Chi‐squared test in both exploratory and validation phases. A *P*‐value<0.05 was considered significant. Wilson score method without continuity correction was used to calculate 95% confidence interval for proportions.^18^ Fleiss' kappa was used for interobserver agreement among the four endoscopists in the validation phase and was interpreted as follows: <0.01 = poor agreement; 0.01–0.20 = slight agreement; 0.21–0.40 = fair agreement; 0.41–0.60 = moderate agreement; 0.61–0.80 = substantial agreement; and 0.81–1.00 = almost perfect agreement.^19^This study and the use of endoscopy images were approved by the Human Research Ethics Committee (TQEH/LMH/MH/2008128) in Australia and by the Nagoya University Hospital Ethics Review Committee (2015–0485) in Japan. This study is presented in accordance to the STROBE statement.^20^

## Results

As per the inclusion and exclusion criteria, a total of 291 patients consented. Of those, 56 were excluded due to intraprocedure exclusion criteria. From the 235 remaining, 25 patients (54 CLs) had insufficient data for assessment (e.g. polyp not able to be retrieved). Furthermore, 26 CLs from 22 patients were not confirmed at histology (e.g. melanosis coli) and were thus excluded. In the validation phase, a set of 20 CLs was chosen from the exploratory phase and 10 CLs were selected from a histology‐correlated Japanese image database. The CLs chosen for the validation phase were evenly distributed among the five types predicted by MS and wJNET.

**Figure 4:**
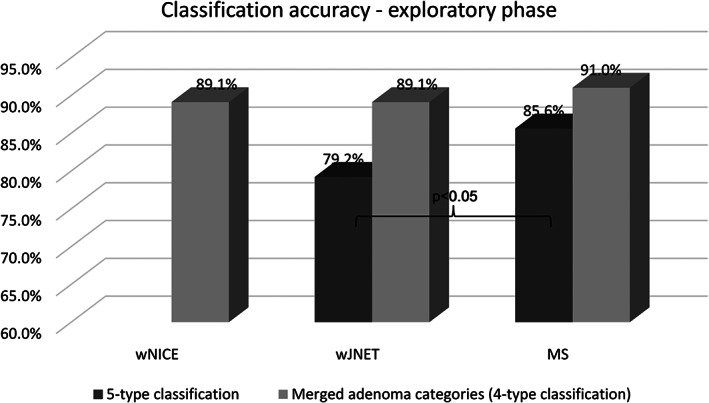
Accuracy for all data with high confidence (exploratory phase).

For the final analysis of the exploratory phase, 188 patients with 483 polyps were evaluated. Most of the evaluated polyps were adenomas (Table 1). Overall, more than 90% were assessed with high confidence by all classifications in the exploratory phase (98.3% with wNICE, 98.3% with wJNET, and 94.8% with MS). For wJNET and MS, the overall accuracies were 78.5% and 83.6%, respectively (*P* = 0.04), while the high‐confidence accuracies were 79.2% and 85.6%, respectively (*P* = 0.01). When early/low‐grade dysplasia (LGD) and advanced/HGD adenoma categories were merged, comparison between wNICE, merged wJNET, and merged MS was made possible and achieved 88.2%, 88.2%, and 88.8% overall accuracy, respectively. For high‐confidence diagnoses, once more, the accuracy was numerically higher for the MS classification, but this difference did not achieve statistical significance (Fig. 4). The subgroup analysis of only diminutive polyps showed similar results to the whole cohort (Table 2). When evaluating the ability to predict neoplastic *versus* nonneoplastic lesions, the accuracy for wNICE, wJNET, and MS all surpassed 90% (Table 3). Although the negative predictive value (NPV) value did not reach 90% with any of the classifications, this analysis was not restricted to the rectosigmoid region.

**Table 1:** Polyp histology and correlation with classifications' type

			Correlation with classifications' type		
Histology—*n* (%)	Exploratory phase	Validation phase^†^	wNICE	wJNET	MS
Hyperplastic	56 (11.6)	24 (20.0)	1	1	I
Tubular adenoma LGD	237 (49.1)	28 (23.3)	2	2A	II
Tubulovillous adenoma LGD	58 (12.0)	8 (6.7)			IIIa
Villous adenoma LGD	3 (0.6)	0 (0)			
Tubular adenoma HGD	5 (1.0)	0 (0)		2B	
Tubulovillous adenoma HGD	15 (3.1)	16 (13.3)			
Villous adenoma HGD	2 (0.4)	0 (0)			
SSA/P no dysplasia	75 (15.5)	24 (20.0)	1 or 2^‡^	1 or 2A^‡^	I/II^‡^ or IIo
SSA/P LGD	8 (1.7)	0 (0.0)			
SSA/P HGD	2 (0.4)	4 (3.3)		2B	IIIa
Superficial cancer	6 (1.2)	0 (0.0)	2		
Invasive cancer	11 (2.3)	16 (13.3)	3	3	IIIb
Other	5 (1.0)	0 (0)	—	—	—

† *n* based on the number of images evaluated by the four endoscopists.

‡ Dependent on serrated features as per the WASP and MS classifications.

HGD, high‐grade dysplasia; LGD, low‐grade dysplasia; MS, modified Sano; SSA/P, sessile serrated adenoma/polyp; WASP, Workgroup serrAted polypS and Polyposis.

**Table 2:** Accuracy of four‐ and five‐type classifications for all data and subsets for exploratory phase

	Overall accuracy % (95% confidence interval)			High‐confidence accuracy % (95% confidence interval)		
Classification	wNICE	wJNET	MODIFIED Sano	wNICE	wJNET	Modified Sano
All data	88.2 (85.0; 90.8)	78.5 (74.6; 81.9)	83.6 (80.0; 86.6)	89.1 (86.0; 91.6)	79.2 (75.3; 82.6)	85.6 (82.1; 88.5)
All data with adenoma categories merged	N/A	88.2 (85.0; 90.8)	88.8 (85.7; 91.3)	N/A	89.1 (86.0; 91.6)	91.0 (88.0; 93.3)
*n*	483	483	483	475	475	458
≤5 mm subset	86.4 (81.4; 90.2)	85.6 (80.6; 89.5)	86.4 (81.4; 90.2)	87.4 (82.5; 91.1)	86.5 (81.5; 90.3)	88.1 (83.1; 91.7)
≤5 mm subset with adenoma categories merged	N/A	86.4 (81.4; 90.2)	86.9 (82.0; 90.6)	N/A	87.4 (82.5; 91.1)	88.6 (83.7; 92.2)
*n*	236	236	236	230	230	219

**Table 3:** Accuracy measures for dichotomy neoplastic *versus* nonneoplastic for high‐confidence diagnosis (exploratory phase)

	Overall diagnosis % (95% confidence interval)			High‐confidence diagnosis % (95% confidence interval)		
Classification	wNICE	wJNET	Modified Sano	wNICE	wJNET	Modified Sano
Accuracy	90.3 (87.3; 92.6)	90.3 (87.3; 92.6)	90.7 (87.8; 93.0)	90.7 (87.8; 93.0)	90.7 (89.0; 94.0)	93.0 (90.3; 95.0)
Sensitivity	95.3 (93.0; 96.9)	95.3 (93.0; 96.9)	96.2 (94.1; 97.6)	95.7 (93.5; 97.2)	95.7 (93.5; 97.2)	98.3 (96.7; 99.2)
Specificity	55.0 (50.5; 59.4)	55.0 (50.5; 59.4)	51.7 (47.3; 56.1)	55.9 (51.4; 60.3)	55.9 (51.4; 60.3)	46.8 (42.3; 51.4)
Positive predictive value	93.7 (91.2; 95.5)	93.7 (91.2; 95.5)	93.3 (90.7; 95.2)	93.9 (91.4; 95.7)	93.9 (91.4; 95.7)	94.2 (91.7; 96.0)
Negative predictive value	62.3 (57.9; 66.5)	62.3 (57.9; 66.5)	66.0 (61.7; 70.1)	64.7 (60.3; 68.9)	64.7 (60.3; 68.9)	75.9 (71.8; 79.6)

The description of misdiagnoses predicted by wNICE, wJNET, and MS classifications are shown in Tables S1–S3 for the exploratory phase and in Tables S4–S6 for the validation phase. In these tables, the misdiagnoses were divided into severe and moderate. Severe misdiagnoses are highlighted in red and were considered when they would have led to a major change in the therapeutic decision (i.e. nonresection of a neoplastic polyp, endoscopic resection of an invasive cancer, or referral for surgery of a noninvasive cancer/benign lesion). Moderate misdiagnoses are highlighted in yellow and were defined as misdiagnoses that might lead to minor therapeutic changes (e.g. resection of a nonneoplastic polyp or resection of a superficially invasive cancer with endoscopic mucosal resection instead of endoscopic submucosal dissection). The color green highlights the correct diagnoses. The rate of severe misdiagnoses for wNICE, wJNET, and MS were 4.4%, 4.4%, and 2.2% for high‐confidence diagnosis, respectively. The rate of moderate misdiagnoses for the same classifications were 6.5%, 16.4%, and 12.2% for high‐confidence diagnosis, respectively. There was a statistically significant difference between wNICE and the other two classifications regarding moderate misdiagnoses alone (*P* < 0.01). This is likely attributed to the inability of the NICE classification to differentiate subtypes of adenomas (e.g. both a low‐grade tubular adenoma and a high‐grade tubulovillous adenoma would be “accurately” classified as a NICE type 2).

**Figure 5:**
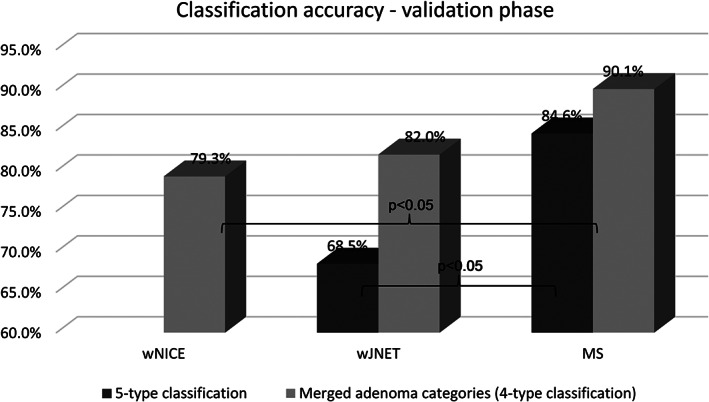
Accuracy for all data with high confidence (validation phase).

In the validation phase, four experienced endoscopists evaluated 30 CL images each. The final dataset of 120 images scored for all classifications consisted of NBI and BLI subsets (80 and 40 images, respectively). Details on histology can be found in Table 1. The results of this phase (high‐confidence accuracy) for the whole dataset and NBI subset confirmed the significantly higher accuracy of MS compared to wJNET found in the exploratory phase (Fig. 5). Accuracy performance for the subset of NBI data can be found in Table 4 and Figure S4.

**Table 4:** Accuracy of four‐ and five‐type classifications for all data and subsets for validation phase

	Overall accuracy % (95% confidence interval)			High‐confidence accuracy % (95% confidence interval)		
Classification	wNICE	wJNET	Modified Sano	wNICE	wJNET	Modified Sano
All data	N/A	70.0 (61.3; 77.5)	81.7 (73.8; 87.6)	N/A	68.5 (59.4; 76.4)	84.6 (75.8; 90.6)
All data with adenoma categories merged	79.2 (71.1; 85.5)	83.3 (75.6; 88.9)	89.2 (82.4; 93.6)	79.3 (70.9; 85.8)	82.0 (73.8; 88.0)	90.1 (82.3; 94.7)
*n*	120	120	120	111	111	91
NBI subset	N/A	65.0 (54.1; 74.6)	77.5 (67.2; 85.3)	N/A	63.0 (51.5; 73.2)	81.4 (69.7; 89.3)
NBI subset with adenoma categories merged	77.5 (67.2; 85.3)	80.0 (70.0; 87.3)	87.5 (78.5; 93.1)	77.5 (66.5; 85.7)	78.1 (67.3; 86.1)	89.8 (79.5; 95.2)
*n*	80	80	80	71	73	59
≤5 mm subset	N/A	67.5 (52.0; 79.9)	80.0 (65.2; 89.5)	N/A	65.7 (49.1; 79.2)	85.7 (68.5; 94.3)
≤5 mm subset with adenoma categories merged	77.5 (62.5; 87.7)	80.0 (65.2; 89.5)	90.0 (77.0; 96.0)	77.8 (61.9; 88.3)	77.1 (60.9; 87.9)	92.9 (77.4; 98.0)
*n*	40	40	40	36	35	28
BLI subset	N/A	80.0 (65.2; 89.5)	90.0 (77.0; 96.0)	N/A	78.9 (63.6; 88.9)	90.6 (75.8; 96.8)
BLI subset with adenoma categories merged	82.5 (68.1; 91.3)	90.0 (77.0; 96.0)	92.5 (80.1; 97.4)	82.5 (68.1; 91.3)	89.5 (75.9; 95.8)	90.6 (75.8; 96.8)
*n*	40	40	40	40	38	32

Interobserver agreement between the four endoscopists in the validation phase achieved substantial agreement for the whole dataset with kappa values of 0.74, 0.69, and 0.63 for NICE, JNET, and MS, respectively. For the high‐confidence subset, the agreement found was almost perfect (*κ* = 0.82), substantial (*κ* = 0.79), and moderate (*κ* = 0.49) for NICE, JNET, and MS, respectively. The variability of results among endoscopists for each classification was not statistically significant.

## Discussion

The MS classification was already shown to have higher accuracy when compared to NICE classification for differentiating neoplastic from nonneoplastic polyps.^10^ However, in this previous study, SSA/Ps diagnosed as type 1 were excluded to mitigate the bias toward MS. This likely impaired the evaluation of MS' true potential. Therefore, in this study we included the WASP classification^13^ as an “add‐on” for an adequate comparison between the current state‐of‐the‐art classifications.

The main outcome was to compare classifications that could both differentiate HPs from SSA/Ps and early from advanced adenomas (i.e. wJNET and MS classifications). The MS classification was the most accurate classification between the two. This was also verified in the external validation phase, which found a higher overall and high‐confidence accuracy for MS compared to wJNET (*P* = 0.04 and *P* < 0.01, respectively). Although a numerically higher accuracy was found for the MS within the BLI subset, this did not reach statistical significance most likely due to the small numbers.

The use of a classification with the ability to differentiate advanced adenomas is important as this may have implications on the resection technique to be used. We hypothesize that the differences found between MS and wJNET were due to how the adenomas are divided within each classification. JNET divides adenomas based on the grade of dysplasia they exhibit (2A = low‐grade dysplasia/low‐grade intramucosal neoplasia and 2B = high‐grade dysplasia/high‐grade intramucosal neoplasia or shallow submucosal invasive cancer). MS, however, separates adenomas based on ‘early’ or ‘advanced adenomas’: tubular adenomas with low‐grade dysplasia—MS II; advanced adenomas (e.g. villous adenomas or tubular adenomas with HGD) are allocated in category IIIa. Our hypothesis is that this slightly different definition may have led to better accuracy results.

In this study, adenoma categories were merged in JNET and MS classifications for adequate comparison with wNICE. This was used as a tool to separately identify the contribution of the WASP criteria to NICE/JNET compared to the MS criteria in differentiating SSA/Ps. Although a slight difference was found in the exploratory phase, a more pronounced difference was found in the validation phase (Fig. 5). The increased accuracy of MS may relate to how the SSA/P criteria differ in each classification. WASP includes four serrated features that are equally considered when characterizing polyps (≥2 features = SSA/P—Figure S3). As per the MS, six serrated features are evaluated where the “open pits” feature is considered sufficient to call a SSA/P with high confidence by itself (Fig. 2). These differences may have led to better results with the MS classification. An interesting subject for future research would be to analyze the performance of the JNET classification when taking into account all six serrated features as per MS, what could be considered a “modified JNET”.

Although the study was validated with external endoscopists and BLI technology, the results might not be representative of all endoscopy centers as accuracy of endoscopic classifications depends on the setting in which it is evaluated.^21^ Nonetheless, we were able to show that MS can potentially extrapolate geographical boundaries and imaging systems. This has also been shown in another study from our group where computer‐aided diagnosis was accurate with both NBI and BLI technologies.^22^ Another study shows the potential of using NBI‐based classifications with BLI technology.^23^ Another limitation to our study is that all CLs were rated for all features/classifications by the same two endoscopists in the same room at the same time, which could lead to bias. An ideal design would have consisted of a larger number of endoscopists in different endoscopy suites. Finally, in our study, we have used distal caps routinely. Although we believe it makes characterization with magnified NBI easier, its use is not obligatory in any of the classifications.

In conclusion, MS can differentiate serrated and adenomatous polyps as a stand‐alone classification. This classification could be beneficial as an ‘all‐encompassing single classification’ rather than using the NICE, JNET, or a combination of them (wNICE, wJNET), which could be impractical and confusing.

## Author Contributions

Leonardo Zorron Cheng Tao Pu and Rajvinder Singh conceptualized and designed the study. Rajvinder Singh was responsible for the study supervision. Leonardo Zorron Cheng Tao Pu, Doreen Siew Ching Koay, and Amanda Ovenden were involved in exploratory phase data extraction. Leonardo Zorron Cheng Tao Pu, Takeshi Yamamura, and Masanao Nakamura were involved in validation‐phase data extraction. Leonardo Zorron Cheng Tao Pu and Suzanne Edwards were involved in the statistical analyses. Leonardo Zorron Cheng Tao Pu, Rajvinder Singh, Alastair D Burt, Mitsuhiro Fujishiro, Yoshiki Hirooka, Masanao Nakamura, and Takeshi Yamamura interpreted the results. Leonardo Zorron Cheng Tao Pu drafted the manuscript. Rajvinder Singh, Alistair D Burt, Masanao Nakamura, Takeshi Yamamura, Yoshiki Hirooka, and Mitsuhiro Fujishiro made critical revisions to the article for important intellectual content. All authors read and approved the final version of the manuscript.

## Supporting information


**Figure S1** JNET classification
**Figure S2** NICE classification
**Figure S3** WASP classification
**Figure S4** Accuracy of NBI subset with high confidence (validation phase)
**Table S1** Diagnoses per histology according to wNICE classification (exploratory phase)
**Table S2** Diagnoses per histology according to wJNET classification (exploratory phase)
**Table S3** Diagnoses per histology according to MS classification (exploratory phase)
**Table S4** High‐confidence diagnoses per type and histology according to wNICE classification at validation phase
**Table S5** High‐confidence diagnoses per type and histology according to wJNET classification at validation phase
**Table S6** High‐confidence diagnoses per type and histology according to MS classification at validation phase
