# Supplementary material for: Comparison of different virtual chromoendoscopy classification systems for the characterization of colorectal lesions
Source: JGH Open. 2020 Jul 7;4(5):818–26. doi: 10.1002/jgh3.12382 (PMC7578300; doi:10.1002/jgh3.12382)
Supplement: Supplementary file 1 — Figure S1 JNET classification Figure S2 NICE classification Figure S3 WASP classification Figure S4 Accuracy of NBI subset with high confidence (validation phase) Table S1 Diagnoses per histology according to wNICE classification (exploratory phase) Table S2 Diagnoses per histology according to wJNET classification (exploratory phase) Table S3 Diagnoses per histology according to MS classification (exploratory phase) Table S4 High‐confidence diagnoses per type and histology according to wNICE classification at validation phase Table S5 High‐confidence diagnoses per type and histology according to wJNET classification at validation phase Table S6 High‐confidence diagnoses per type and histology according to MS classification at validation phase [file JGH3-4-818-s001.docx]

Appendices (Supplementary material)

**Supplementary figure 1 -** JNET classification


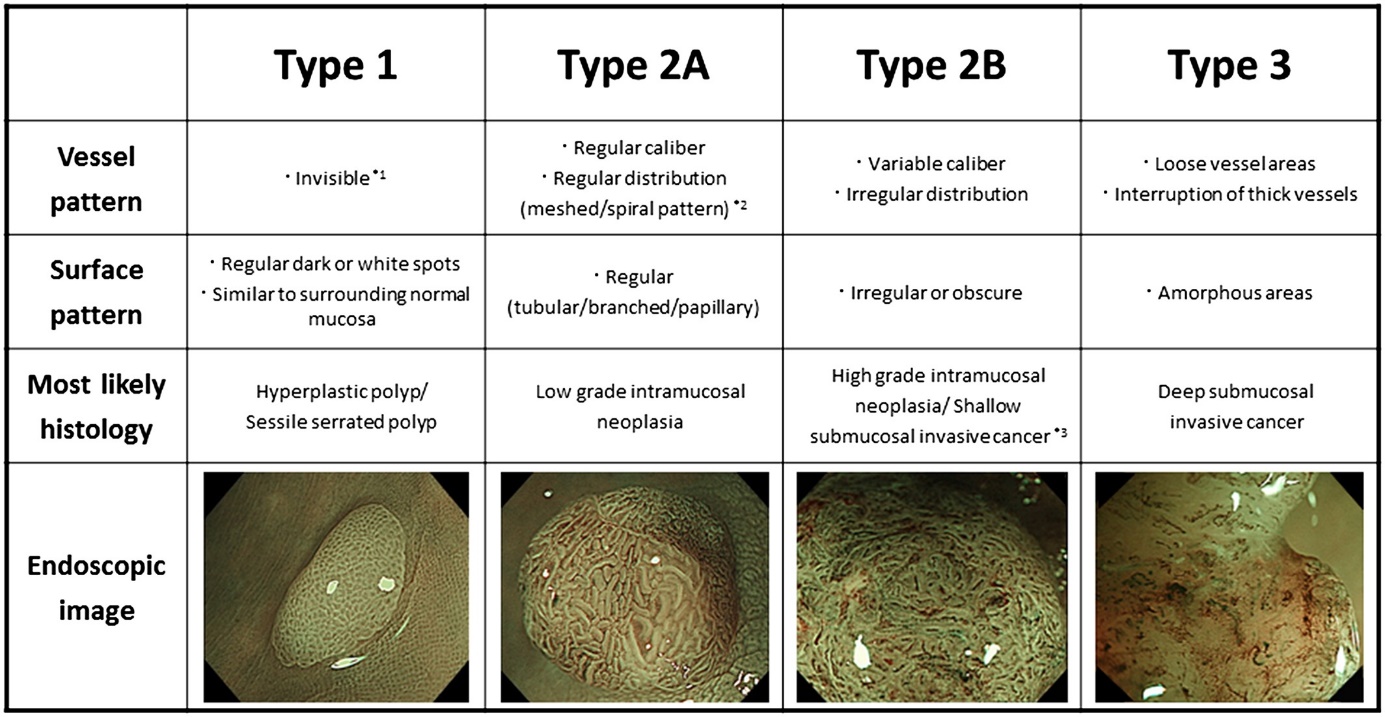


**Supplementary figure 2 -** NICE classification

**
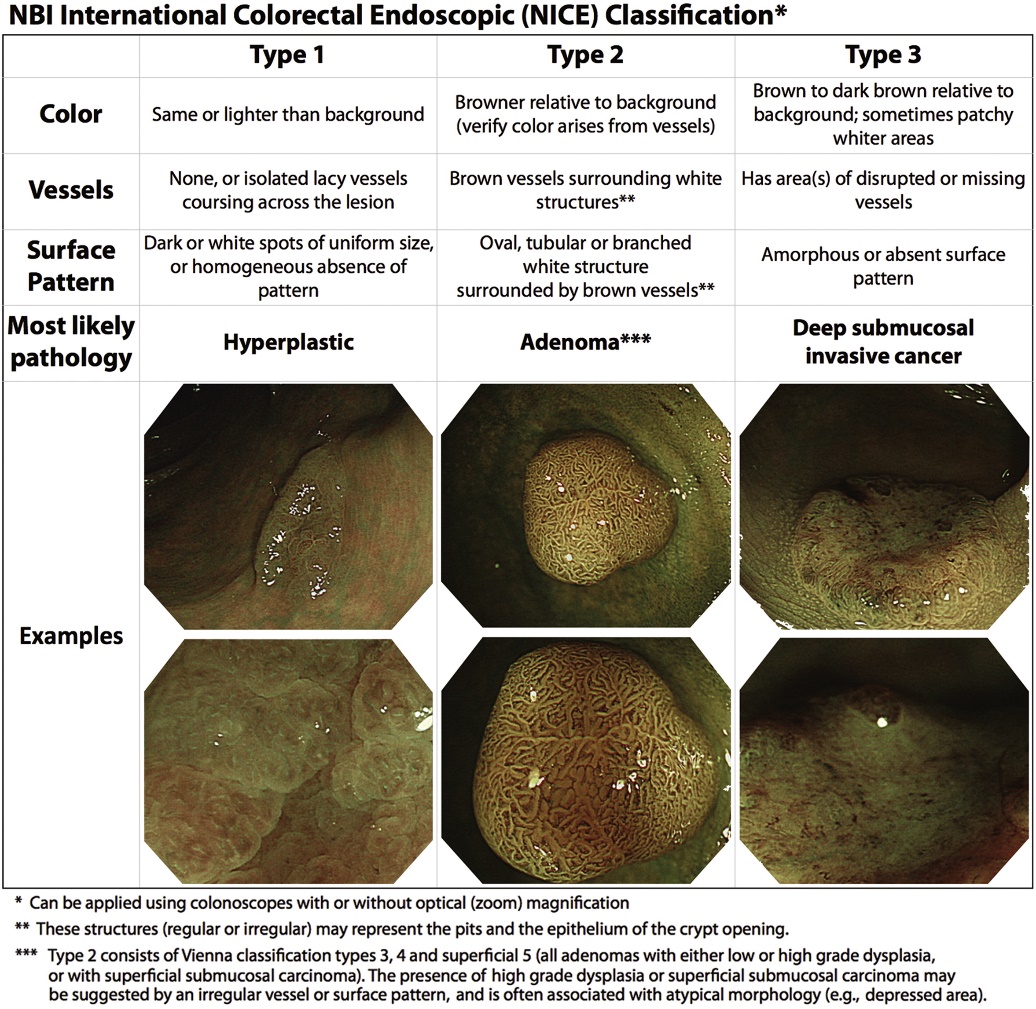
**

**Supplementary figure 3 -** WASP classification


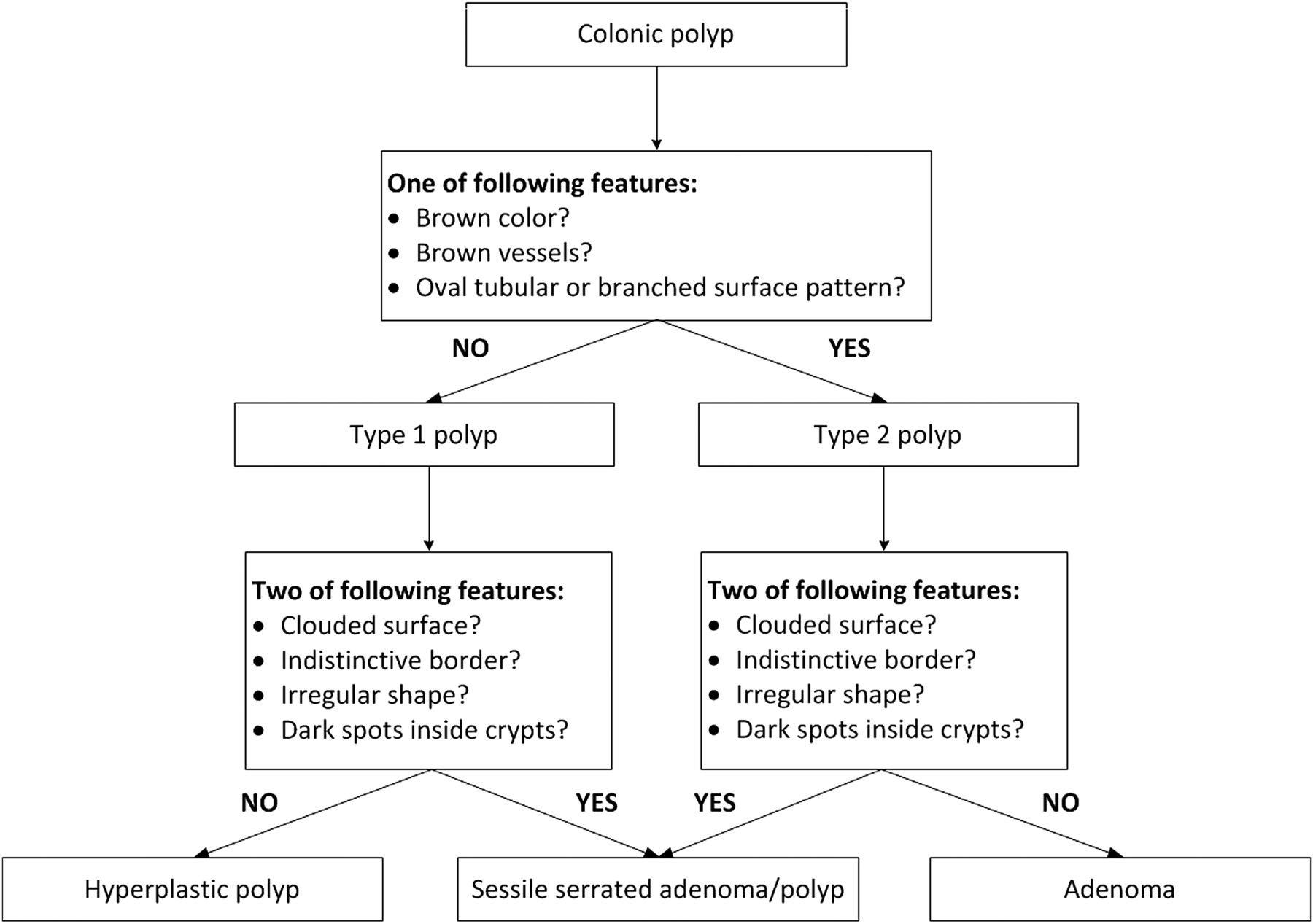


**Supplementary figure 4 –** Accuracy for NBI subset with high confidence (validation phase)

**
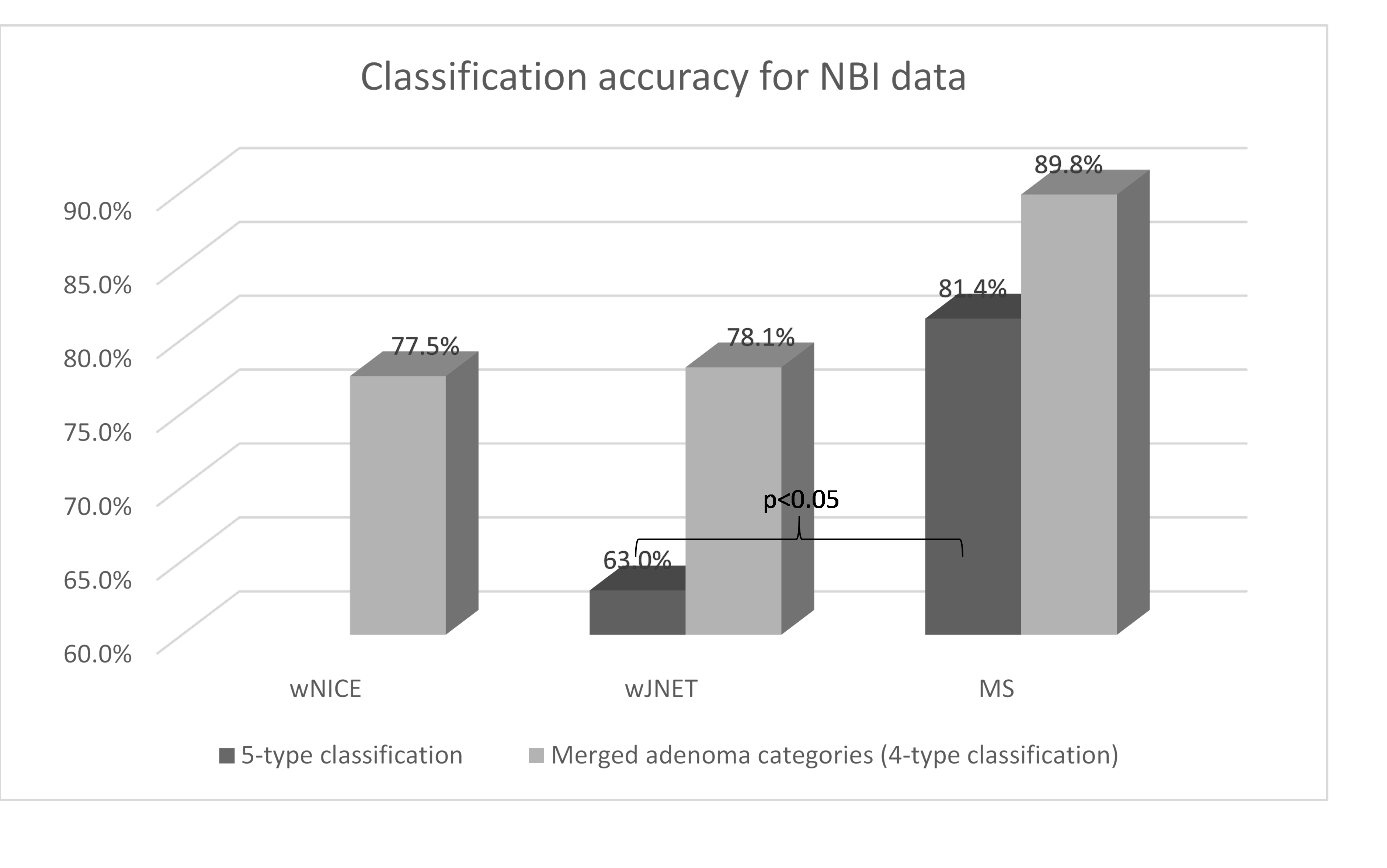
**

**Supplementary Table 1 –** Diagnoses per histology according to wNICE classification (exploratory phase)

|  | **High-confidence diagnosis**  Classification type  (predicted histology) | Histology | | | | | | | | | |
| --- | --- | --- | --- | --- | --- | --- | --- | --- | --- | --- | --- |
|  |  | HP | Inflammatory | SSA/P up to LGD | TA LGD | TVA or VA LGD | | TSA LGD | SSA/P HGD | Adenoma HGD/ Superficial cancer | Invasive cancer |
| **wNICE classification** | wNICE 1 (HP) | 33 | 0 | 14 | 4 | | 0 | 0 | 0 | 0 | 0 |
|  | wNICE 1 (SSA/P up to LGD) | 9 | 0 | 61 | 2 | | 0 | 0 | 0 | 0 | 0 |
|  | wNICE 2 (SSA/P up to LGD) | 2 | 0 | 1 | 0 | | 0 | 0 | 0 | 0 | 0 |
|  | wNICE 2  (adenoma/HGD/superficial cancer) | 11 | 4 | 3 | 228 | | 61 | 1 | 2 | 28 | 3 |
|  | wNICE 3 (invasive cancer) | 0 | 0 | 0 | 0 | | 0 | 0 | 0 | 0 | 8 |

Legend:

| Severe misdiagnosis |
| --- |
| Moderate misdiagnosis |
| Accurate diagnosis |

HP – Hyperplastic polyp

SSA/P – Sessile serrated adenoma/polyp

TA – Tubular adenoma

TVA – Tubulovillous adenoma

VA – Villous adenoma

TSA – Traditional serrated adenoma

LGD – Low grade dysplasia

HGD – High grade dysplasia

**Supplementary Table 2 -** Diagnoses per histology according to wJNET classification (exploratory phase)

|  | **High-confidence diagnosis**  Classification type  (predicted histology) | Histology | | | | | | | | |
| --- | --- | --- | --- | --- | --- | --- | --- | --- | --- | --- |
|  |  | HP | Inflammatory | SSA/P up to LGD | TA LGD | TVA or VA LGD | TSA LGD | SSA/P HGD | Adenoma HGD/ Superficial cancer | Invasive cancer |
| **wJNET classification** | wJNET 1 (HP) | 33 | 0 | 14 | 4 | 0 | 0 | 0 | 0 | 0 |
|  | wJNET 1 (SSA/P up to LGD) | 9 | 0 | 61 | 2 | 0 | 0 | 0 | 0 | 0 |
|  | wJNET 2A (SSA/P up to LGD) | 2 | 0 | 1 | 0 | 0 | 0 | 0 | 0 | 0 |
|  | wJNET 2A  (adenoma LGD) | 11 | 4 | 3 | 222 | 26 | 0 | 1 | 4 | 0 |
|  | wJNET 2B  (adenoma/HGD/superficial cancer) | 0 | 0 | 0 | 6 | 35 | 1 | 1 | 24 | 3 |
|  | wNICE 3 (invasive cancer) | 0 | 0 | 0 | 0 | 0 | 0 | 0 | 0 | 8 |

Legend:

| Severe misdiagnosis |
| --- |
| Moderate misdiagnosis |
| Accurate diagnosis |

HP – Hyperplastic polyp

SSA/P – Sessile serrated adenoma/polyp

TA – Tubular adenoma

TVA – Tubulovillous adenoma

VA – Villous adenoma

TSA – Traditional serrated adenoma

LGD – Low grade dysplasia

HGD – High grade dysplasia

**Supplementary Table 3 -** Diagnoses per histology according to MS classification (exploratory phase)

|  | **High-confidence diagnosis**  Classification type  (predicted histology) | Histology | | | | | | | | |
| --- | --- | --- | --- | --- | --- | --- | --- | --- | --- | --- |
|  |  | HP | Inflammatory | SSA/P up to LGD | TA LGD | TVA or VA LGD | TSA LGD | SSA/P HGD | Adenoma HGD/ Superficial cancer | Invasive cancer |
| **MS classification** | MS I (HP) | 22 | 0 | 3 | 4 | 0 | 0 | 0 | 0 | 0 |
|  | MS IIo (SSA/P up to LGD) | 10 | 0 | 64 | 2 | 0 | 0 | 0 | 0 | 0 |
|  | MS II (TA LGD) | 10 | 2 | 4 | 221 | 11 | 0 | 0 | 4 | 0 |
|  | MS IIIa  (TVA/VA/HGD/superficial cancer) | 1 | 2 | 0 | 10 | 50 | 1 | 2 | 24 | 3 |
|  | MS IIIb (invasive cancer) | 0 | 0 | 0 | 0 | 0 | 0 | 0 | 0 | 8 |

Legend:

| Severe misdiagnosis |
| --- |
| Moderate misdiagnosis |
| Accurate diagnosis |

HP – Hyperplastic polyp

SSA/P – Sessile serrated adenoma/polyp

TA – Tubular adenoma

TVA – Tubulovillous adenoma

VA – Villous adenoma

TSA – Traditional serrated adenoma

LGD – Low grade dysplasia

HGD – High grade dysplasia

**Supplementary Table 4 –** High-confidence diagnoses per type and histology according to wNICE classification at validation phase

|  | **High confidence diagnosis**  Classification type  (predicted histology) | Histology | | | | | | | | | |
| --- | --- | --- | --- | --- | --- | --- | --- | --- | --- | --- | --- |
|  |  | HP | Inflammatory | SSA/P up to LGD | TA LGD | TVA or VA LGD | | TSA LGD | SSA/P HGD | Adenoma HGD/ Superficial cancer | Invasive cancer |
| **wNICE classification** | wNICE 1 (HP) | 19 | 0 | 8 | 1 | | 0 | 0 | 0 | 0 | 1 |
|  | wNICE 1 (SSA/P up to LGD) | 5 | 0 | 15 | 0 | | 1 | 0 | 0 | 0 | 0 |
|  | wNICE 2 (SSA/P up to LGD) | 0 | 0 | 0 | 1 | | 0 | 0 | 0 | 0 | 0 |
|  | wNICE 2  (adenoma/HGD/superficial cancer) | 0 | 0 | 0 | 23 | | 6 | 0 | 3 | 12 | 2 |
|  | wNICE 3 (invasive cancer) | 0 | 0 | 0 | 0 | | 0 | 0 | 0 | 4 | 10 |

Legend:

| Severe misdiagnosis |
| --- |
| Moderate misdiagnosis |
| Accurate diagnosis |

HP – Hyperplastic polyp

SSA/P – Sessile serrated adenoma/polyp

TA – Tubular adenoma

TVA – Tubulovillous adenoma

VA – Villous adenoma

TSA – Traditional serrated adenoma

LGD – Low grade dysplasia

HGD – High grade dysplasia

**Supplementary Table 5 –** High-confidence diagnoses per type and histology according to wJNET classification at validation phase

|  | **High confidence diagnosis**  Classification type  (predicted histology) | Histology | | | | | | | | |
| --- | --- | --- | --- | --- | --- | --- | --- | --- | --- | --- |
|  |  | HP | Inflammatory | SSA/P up to LGD | TA LGD | TVA or VA LGD | TSA LGD | SSA/P HGD | Adenoma HGD/ Superficial cancer | Invasive cancer |
| **wJNET classification** | wJNET 1 (HP) | 19 | 0 | 8 | 1 | 0 | 0 | 0 | 0 | 1 |
|  | wJNET 1 (SSA/P up to LGD) | 5 | 0 | 15 | 0 | 2 | 0 | 0 | 0 | 0 |
|  | wJNET 2A (SSA/P up to LGD) | 0 | 0 | 0 | 0 | 0 | 0 | 0 | 0 | 0 |
|  | wJNET 2A  (adenoma LGD) | 0 | 0 | 0 | 20 | 4 | 0 | 1 | 10 | 0 |
|  | wJNET 2B  (adenoma/HGD/superficial cancer) | 0 | 0 | 0 | 3 | 1 | 0 | 2 | 6 | 3 |
|  | wNICE 3 (invasive cancer) | 0 | 0 | 0 | 0 | 0 | 0 | 0 | 0 | 10 |

Legend:

| Severe misdiagnosis |
| --- |
| Moderate misdiagnosis |
| Accurate diagnosis |

HP – Hyperplastic polyp

SSA/P – Sessile serrated adenoma/polyp

TA – Tubular adenoma

TVA – Tubulovillous adenoma

VA – Villous adenoma

TSA – Traditional serrated adenoma

LGD – Low grade dysplasia

HGD – High grade dysplasia

**Supplementary Table 6 –** High-confidence diagnoses per type and histology according to MS classification at validation phase

|  | **High confidence diagnosis**  Classification type  (predicted histology) | Histology | | | | | | | | |
| --- | --- | --- | --- | --- | --- | --- | --- | --- | --- | --- |
|  |  | HP | Inflammatory | SSA/P up to LGD | TA LGD | TVA or VA LGD | TSA LGD | SSA/P HGD | Adenoma HGD/ Superficial cancer | Invasive cancer |
| **MS classification** | MS I (HP) | 11 | 0 | 0 | 0 | 0 | 0 | 0 | 0 | 0 |
|  | MS IIo (SSA/P up to LGD) | 3 | 0 | 23 | 0 | 2 | 0 | 0 | 0 | 1 |
|  | MS II (TA LGD) | 0 | 0 | 0 | 17 | 1 | 0 | 0 | 0 | 0 |
|  | MS IIIa  (TVA/VA/HGD/superficial cancer) | 0 | 0 | 0 | 4 | 3 | 0 | 2 | 12 | 2 |
|  | MS IIIb (invasive cancer) | 0 | 0 | 0 | 0 | 0 | 0 | 0 | 1 | 9 |

Legend:

| Severe misdiagnosis |
| --- |
| Moderate misdiagnosis |
| Accurate diagnosis |

HP – Hyperplastic polyp

SSA/P – Sessile serrated adenoma/polyp

TA – Tubular adenoma

TVA – Tubulovillous adenoma

VA – Villous adenoma

TSA – Traditional serrated adenoma

LGD – Low grade dysplasia

HGD – High grade dysplasia
